# Interstitial Glucose and Physical Exercise in Type 1 Diabetes: Integrative Physiology, Technology, and the Gap In-Between

**DOI:** 10.3390/nu10010093

**Published:** 2018-01-15

**Authors:** Othmar Moser, Jane E. Yardley, Richard M. Bracken

**Affiliations:** 1 Diabetes Research Group, Medical School, Swansea University, Swansea SA2 8PP, UK; othmar.moser@swansea.ac.uk; 2 Applied Sport, Technology, Exercise and Medicine Research Centre (A-STEM), College of Engineering, Swansea University, Swansea SA1 8EN, UK; 3 Kinesiology, Sport and Recreation, Social Sciences, University of Alberta, Edmonton, AB T4V 2R3, Canada; jane.yardley@ualberta.ca

**Keywords:** continuous glucose monitoring, flash glucose monitoring, exercise, interstitium

## Abstract

Continuous and flash glucose monitoring systems measure interstitial fluid glucose concentrations within a body compartment that is dramatically altered by posture and is responsive to the physiological and metabolic changes that enable exercise performance in individuals with type 1 diabetes. Body fluid redistribution within the interstitial compartment, alterations in interstitial fluid volume, changes in rate and direction of fluid flow between the vasculature, interstitium and lymphatics, as well as alterations in the rate of glucose production and uptake by exercising tissues, make for caution when interpreting device read-outs in a rapidly changing internal environment during acute exercise. We present an understanding of the physiological and metabolic changes taking place with acute exercise and detail the blood and interstitial glucose responses with different forms of exercise, namely sustained endurance, high-intensity, and strength exercises in individuals with type 1 diabetes. Further, we detail novel technical information on currently available patient devices. As more health services and insurance companies advocate their use, understanding continuous and flash glucose monitoring for its strengths and limitations may offer more confidence for patients aiming to manage glycemia around exercise.

## 1. Introduction

Type 1 diabetes is characterized by the autoimmune destruction of pancreatic β-cells within the islets of Langerhans and loss of endogenous insulin production. Most diagnoses are made early in life and result in a life-long dependency on pharmacological insulin, carbohydrate counting, and frequent blood glucose measurements to manage blood glucose levels [1]. In the United Kingdom, over a quarter of a million people have type 1 diabetes and this number is rising. The cost to the National Health Service (NHS) for treatment and care is approximately ₤1.8+ billion a year [2]. Low levels of physical activity are evident in people with type 1 diabetes [3] and large cohort studies demonstrate an increased risk of cardiovascular disease [4,5]; however, regular physical activity is advocated [6,7] and conveys important health benefits [8]. Nevertheless, the increased risk of developing hypoglycemia and the loss of glucose control around exercise are major concerns for people with type 1 diabetes, contributing to the failure to initiate or maintain regular physical activity [9].

Frequent finger prick blood sampling and adjustments in exogenous basal and/or bolus insulin and carbohydrate intake are necessary to minimize glycemic disturbances with physical exercise [6,7]. However, mimicking the natural secretory pattern of endogenous insulin in individuals without diabetes whilst maintaining glycemia within a normal physiological range is challenging around exercise [6,10]. In the Diabetes Control and Complications Trial, intensive treatment to maintain euglycemia between 4 and 7 mmol/L with either an external insulin pump or three or more daily insulin injections and frequent blood glucose measurements delayed the onset and slowed the progression of retinopathy, nephropathy, and neuropathy compared with conventional therapy of one or two daily insulin injections over a 6.5-year period [11,12,13,14]. However, an increased occurrence of severe hypoglycemia was evident with intensive treatment compared to the conventional arm [15]. Thus, more frequent finger prick sampling is inconvenient for patients, increases cost to healthcare providers, and might still not stabilize glycemic control.

In recent years, devices that monitor glucose levels continuously offer opportunities for painless observations of glucose that may guide better glucose management in the daily life of people with type 1 diabetes [16]. Emerging work now demonstrates longitudinal improvements in glucose management with the use of continuous glucose monitoring systems in the daily life of people with type 1 diabetes [17]. Flash glucose monitoring systems, similar to continuous glucose monitoring systems, are now available on prescription in the UK NHS for patients with type 1 diabetes. Current devices monitor dynamic interstitial glucose information for up to two weeks without the need for confirmatory finger prick blood glucose measures. However, whilst mean absolute relative differences demonstrate strong similarity to reference blood values [18], these data are generally reported during rest and not during dynamic exercise.

Performing an acute bout of exercise evokes rapid changes in many physiological systems and demands constant adjustment to facilitate glucose provision to the exercising tissues in both healthy individuals and people with type 1 diabetes [10,19]. The net exercise-induced glucose response in the circulation results from changes in carbohydrate ingestion, hepatic release and uptake, skeletal muscle and adipose tissue uptake (amongst other organs), or hepatic gluconeogenesis, and is dependent on the exercise characteristics: type, mode, intensity, and duration [20,21,22,23]. Importantly, (and often overlooked) acute exercise also causes significant redistribution in body fluid between the interstitium, lymphatics, and bloodstream [24]. Changes in posture, sweating, muscle contraction, and hydration dramatically impact interstitial fluid volume and result in altered concentrations of glucose due to fluid redistribution within this compartment.

This review does not seek to revise the historical development of interstitial glucose monitoring technology or its inclusion around physical activity *per se* but rather seeks to focus on our current understanding of acute physiological changes around exercise involving the interstitium and device performance around acute physical exercise in individuals with type 1 diabetes. A firm understanding of the complex physiological adaptations to acute exercise may inform the reader of the strengths and limitations in tracking glucose using continuous and flash monitoring systems in a dynamic metabolic environment such as exercise and offer more confidence for people with type 1 diabetes to better manage glycemia around exercise.

## 2. Physiological Adjustments to Increased Blood Flow and Glucose Provision during Physical Exercise

### 2.1. Initiation of Physical Exercise

Complex physiological adaptations dictate the ability of skeletal muscle to generate and maintain power in healthy individuals and people with type 1 diabetes [25,26]. The cardiorespiratory system plays a critical role in facilitating increased provision of glucose and oxygen (alongside the removal of carbon dioxide and other metabolic by-products) to exercising muscle. Yet, physical exercise begins with mental activity! In the brain, increased nerve impulses from the cerebral cortex act on the medulla oblongata and suppress vagal tone to stimulate an increase in cardiac output (i.e., heart rate × stroke volume) from ~5 L/min at rest to over 25 L/min in individuals with type 1 diabetes [27]. Central brain areas also raise the baroreflex level to allow blood pressure regulation at a higher set-point, which facilitates higher sympathoadrenal activity and allows further increases in cardiac output. Skeletal muscle arterioles dilate via the actions of increased circulating catecholamines and sympathetic nerve activity on vascular β-adrenoceptors [20]. In non-active splanchnic and renal beds, a greater vascular constriction occurs to allow more blood to flow to exercising areas [27].

### 2.2. Continuation of Physical Exercise

As exercise continues, cardiovascular and respiratory systems continuously adjust to the exercise workload to ensure glucose- and oxygen (O_2_)-rich blood reaches working muscles at the required rate. Elevated cerebral motor center activity and skeletal muscle afferent fiber mechanical (tension) and metabolic (e.g., H^+^, K^+^, and local ischemia) signals stimulate sympathetic nervous system activity and causes increases in circulating catecholamines that act on adrenoceptors on vessels and skeletal muscle cells and consequently vasodilation in healthy individuals [28]. Local to the exercising muscle and heart, metabolites cause vasodilation of skeletal muscle and coronary arterioles. This exercise-induced hyperemia or ‘exercise pressor reflex’ decreases vascular resistance and increases blood flow concomitant with increased capillary recruitment [29]. In high-intensity activities such as sprinting or strength exercises that involve a Valsalva-type maneuver, vascular compression and decreased blood flow induce the buildup of metabolites that activate chemoreceptors, resulting in a greater exercise pressor reflex than dynamic activities, and cause increased cardiac output, total peripheral resistance, and tachycardia that alters blood flow rate in healthy individuals [29]. Increased sympathoadrenal activity constricts arterioles in the non-exercising areas. Also, exercise-induced sympathoadrenal activity increases suppression of vagal activity proportional to the exercise intensity and vasoconstriction of the smooth muscle arterioles. Cutaneous blood flow is initially reduced via vasoconstriction but then increases as vasodilatation occurs due to a rise in body temperature to promote cooling [20]. However, with long duration high-intensity exercise, cutaneous perfusion may again fall as vasoconstriction re-diverts blood flow to muscle. Brain blood flow tends to remain largely constant. During inspiration, pressure in the thoracic cavity falls. With diaphragm contraction during inspiration, there is an increased abdominal pressure that compresses veins and venous blood flow in the abdomen and drives blood back to the heart [30]. The thoracoabdominal pump is also appreciable during heavy exercise as tidal volume and breath frequency increase ventilation rates to over 150 L/min, which was observed in healthy and diseased individuals.

### 2.3. Blood Flow Distribution to Skeletal Muscle during Exercise

At rest, around two-thirds of the blood volume is present in veins and most capillaries are closed; blood flow rates to skeletal muscle is approximately 20–30 mL·min^−1^·kg muscle^−1^ at an open capillary density of ~100 capillaries per mm^2^ in healthy individuals [31]. During exercise, open capillaries increase five-fold due to vasodilation from sympathoadrenal activity and locally from factors emanating from skeletal muscle metabolism (decrease in pO_2_, rise in pCO_2_, decreased pH, temperature, adenosine, NO, P_i_, K^+^). Thus, increased cardiac output and localized vasodilation together account for up to a 100-fold increase in blood flow to working tissues (2–3 L·min^−1^·kg muscle^−1^ during heavy exercise). Further, to facilitate this increased rate of blood flow, a rapid shunting of venous blood to the arterial circuit occurs by vasoconstriction of major veins.

Notwithstanding rapid blood redistribution within the circulation, plasma volume may fall ~20% (1 L) with intense exercise over 15 min due to fluid movement towards active muscle [28]. Alterations in the osmolarity of skeletal myocyte cytoplasm and interstitial fluid due to local release of metabolic factors from skeletal muscle increase solutes in the interstitial fluid [28]. Also, an increased perfusion of capillary beds in active muscle increases capillary hydrostatic pressure, which drives more fluid from the plasma to the interstitium [20]. The extent of plasma volume reduction can be magnified by the ambient temperature and humidity and offset by oral consumption of fluids. The overall effect of a net loss in plasma volume is an apparent increase in red blood cell count and hemoglobin concentration, which increases the O_2_-carrying capacity per liter of blood, but at the expense of a reduction in total blood volume and an increase in viscosity. The movement of fluid from the plasma to the interstitium is offset to a certain extent by fluid movement back into the circulation from tissues that undergo vasoconstriction (gut, kidneys) [32].

## 3. The Interstitial Compartment and Factors Influencing Movement of Interstitial Fluid during Exercise

The interstitial space lies between the circulatory system and organs such as skeletal muscle, myocardium, and liver and contains approximately three-quarters of the body’s total extracellular fluid (e.g., ~10–12 L in a 70-kg person) [33]. Plasma and lymph fluid represent most of the remaining quarter (~3 L) of extracellular fluid. Interstitial fluid represents a quarter of total body water mixing with plasma, lymph fluid, and sweat [33].

The factors that determine the speed and magnitude of movement of fluid between capillaries and interstitium are determined by Starling forces [34]. The net filtration pressure (NFP) is determined by two pressure differentials that determine the net drive for directional fluid movement between the capillary and interstitial space, namely:

(i) Colloid osmotic pressure between capillary (P_c_) and interstitial space (P_if_)

Capillary colloidal pressure is exerted by proteins such as albumin, which causes fluid to be pulled back into the capillary at the venous end due to a higher solute concentration [35]. The remaining fluid left in the interstitial space is drained by the lymphatic capillaries and returned to the circulation. The interstitial colloidal pressure is regulated by the interstitial fluid concentration and the permeability of the capillary to different proteins. As plasma contains more protein than the interstitial fluid, capillary colloidal pressure tends to dominate all the way along the capillary wall [35].

(ii) Hydrostatic pressures from capillary (π_c_) to interstitial space (π_if_)

Colloidal hydrostatic pressure is exerted by the plasma fluid against the capillary wall, moving the fluid into the interstitium through filtration and is greater at the arterial end of a capillary due to the higher blood pressure [36]. The interstitial hydrostatic pressure is determined by the interstitial fluid volume and the surrounding tissues’ compliance with changes in volume and pressure; skin and skeletal muscle have higher compliance to changes in volume and pressure with more soft tissue surrounding them than the brain [37].

Thus, net filtration pressure (NFP) = P_c_ + π_if_ − P_if_ − π_c_ and the transendothelial fluid filtration rate (cm^3^/s) is represented by Equation (1):

J_v_ = L_p_*S* [(P_c_ − P_if_) − σ(π_c_ − π_if_)] (1)

where

- L_p_ is the hydraulic conductivity (which provides a measure of water permeability; m^2^·s·kg^−1^ or m·s^−1^·mmHg^−1^);
- *S* is the surface area available for fluid exchange in m^2^;
- σ is the osmotic reflection coefficient (dimensionless unit) where σ close to 1.0 indicates the capillary is fully effective in allowing fluid and smaller solutes to filter to the interstitial space while larger protein molecules such as albumin are retained. Where σ is <1.0, capillary filter function is reduced.

Acute physical exercise does not alter the characteristics of the microvascular exchange vessels [38,39,40]. Thus, *S* (the surface area available for fluid exchange) contributes most to the enhanced filtration rates with acute exercise but alterations in hydraulic conductivity (L_p_) and/or the osmotic reflection coefficient (σ) do not.

Although most pressure fluctuations are absorbed at the level of precapillary resistance vessels, even modest increases in capillary filtration drive lead to fluid accumulation in the interstitial compartment and can increase from 0.008–0.015 mL/min/mmHg for resting skeletal muscle to greater than 4-fold during exercise in healthy individuals [24]. Thus, the rate of fluid filtration from the blood to the tissue interstitial space can reach 1.5 mL·min^−1^·100 g muscle^−1^ during intense rhythmic contraction [39,40,41,42]. This rapid rate of fluid movement with exercise initially exceeds the drainage capacity of the lymphatic system and, converse to the loss of plasma fluid, is a gain in interstitial fluid volume of ~15–20% in exercising tissue [43]. This is due to several factors: (i) exercise-induced capillary hydrostatic pressure increasing with arteriolar vasodilation [43]; (ii) relaxation of vascular smooth muscle in arterioles increasing capillary surface area by recruiting capillaries that were previously closed [41]; and (iii) a muscular contraction-induced increase in interstitial fluid osmolarity due to released metabolites (e.g., K^+^, lactate, H^+^, adenosine, ammonia) from active skeletal muscle cells that transiently raises interstitial fluid osmolarity by ~20–30 mmol/L or 7–10% [41]. Also, an expanded interstitial fluid volume increases the diffusion distance for glucose molecules and may hinder cellular metabolism in swollen tissues. Interstitial (or cellular) edema can also impair glucose tissue perfusion by collapsing capillaries in swollen tissue [44].

The initial rate of hypervolemia in the interstitium slows as exercise continues due to (i) a compensatory absorption of interstitial fluid by vasoconstricted vascular beds where capillary pressure is lower in non-exercising tissues; and (ii) the subsequent reduction in flow rate allows better lymphatic drainage [43] with one-way flow [44] and flow-induced lymphatic dilation. Muscle contraction increases interstitial fluid pressure and reduces the transcapillary hydrostatic gradient, further reducing fluid accumulation with sustained exercise [45].

## 4. Interstitial Glucose Metabolism during Exercise

The small amount of glucose in the interstitium of an adult individual with type 1 diabetes (e.g., 10 mmol/L = 1.8 g/L = 18 g with an interstitial fluid volume of 10 L) represents the point measurement of glucose changes at that measured site. Patients in a fasted, resting state produce small rate changes in the disappearance and/or appearance of glucose. However, acute exercise dramatically increases carbohydrate combustion and the metabolic turnover [46] of glucose increases 40 to 50-fold in exercising tissues (Table 1) [33,47]. The magnitude of change in interstitial glucose with acute physical exercise in people with type 1 diabetes is dependent on the type, mode, intensity, and duration of exercise, as well as the time of day, circulating insulin levels, and whether the individual is fasted or fed.

### 4.1. Glucose Transport to Exercising Muscle

Capillary recruitment shortens the diffusion distance and increases the surface area, with the diffusional capacity increased further by an effect of blood flow on permeability. A fall in tissue glucose concentrations raises the gradient across the capillary wall and the increase in diffusional flux raises the fractional extraction and arteriovenous concentration difference (*C_a_*-*C_v_*) [48]. An increased blood flow delivers glucose faster to the capillary and prevents a major fall in the mean intra-capillary plasma concentration, thereby avoiding a flow-limitation of exchange. The new trans-capillary glucose flux is, thus, determined from the Fick principle equaling blood flow × increased arteriovenous concentration difference (Table 1) [48].

### 4.2. Exercising Skeletal Muscle Tissue Is a Glucose Consumer; Inactive Tissue Acts as a Glucose Store

Glucose entry into muscle occurs across the cell membrane via the glucose transporter type-4 (GLUT-4) [49]. Once inside the muscle, glucose is irreversibly converted to glucose 6-phosphate via the enzyme hexokinase to prevent loss of this valuable nutrient from muscle and is either utilized via glycolysis or stored as glycogen until needed [49]. Skeletal muscle glycogen stores are about 300–350 g and can be manipulated with low carbohydrate diets to reduce estimated whole-body stores from 300 to 50 mmol·glucosyl units per kg^−1^ wet weight in healthy individuals [50]. Conversely, short-term high carbohydrate diets markedly increase muscle glycogen stores to more than 500 mmol·glucosyl units per kg^−1^ wet weight. Carbohydrate combustion in the absence of oxygen is important for releasing energy during high-intensity exercises such as sprinting or strength exercises. For example, a 6 second cycle sprint reduces quadriceps muscle glycogen concentration by 14% from 316 ± 75 to 273 ± 80 mmol·glucosyl units per kg^−1^ wet weight [51] and by 32% after a 30 second sprint [21]. Consequently, blood lactate and H+ concentrations increase from ~1.0 to over 20 mmol/L and 7.4 to <7 pH units and alters osmolarity in the interstitial space [21,51]. The net glucose uptake into tissues during exercise depends on the exercise characteristics; moderate intensity exercise causes similar whole-body rates of glucose appearance and disappearance. In contrast, high-intensity exercise causes a disproportionate rate of increase in glucose appearance compared to disappearance [52].

### 4.3. Hepatocytes Take Up and Release Glucose

The liver glycogen content is approximately 80–110 g for a 70-kg person and similar to muscle is easily manipulated with low carbohydrate diets, reducing hepatic stores from 232 to 24–55 mmol·glucosyl units per kg^−1^ wet weight within 24 h in healthy people [53]. Conversely, high carbohydrate diets markedly increase hepatic glycogen stores to supernormal values of 424–624 mmol·glucosyl units per kg^−1^ wet weight [53], especially if preceded by glycogen-depleting exercise, a concept known as glycogen supercompensation [50]. In contrast to skeletal myocytes, hepatocytes contain a phosphatase enzyme that catalyzes stored glucose, allowing the glucose to leave the liver cell and enter the interstitial space prior to appearing in the circulation. A strong counter-regulatory hormone response elicited by high-intensity exercise acts on hepatocytes to promote hepatic glucose release and consequent exercise-induced hyperglycemia in people with type 1 diabetes [54]. Carbohydrate combustion in the presence of oxygen can increase from resting values of ~0.25 g/min to over 3.0 g/min and the liver plays an important role in the later stages of endurance exercise to support exercise metabolism beyond that of lipid [20]. On the other hand, one day of starvation and/or prolonged exercise might almost deplete endogenous carbohydrate stores, excepting for the body’s ability to minimize this loss through the manufacture of new glucose from amino acids, ketoacids, or lactate in the liver; the contribution of gluconeogenesis to exercise is important [20,22].

### 4.4. Exogenous ‘On-Board’ Insulin Concentrations Differ When Compared with Endogenous Insulin

In people that do not have diabetes, plasma insulin falls with exercise duration and/or intensity due to exercise-induced catecholamine inhibition on pancreatic ß-cell function [55]. However, no such reductions on exogenous insulin are seen in individuals with type 1 diabetes. Indeed, there is evidence of increased circulating insulin with exercise [56]. Insulin stimulates vasodilation around subcutaneous depots where injected insulin may be flushed into the extracellular space and increase glucose uptake and hepatic glucose inhibition [57].

### 4.5. Ingested Carbohydrate from the Gastrointestinal Tract

Ingested carbohydrates (e.g., a mixture of glucose and fructose) can be assimilated from the small intestine at an upper limit of ~1.8 g/min [58]. Interestingly, glucose alone can be absorbed and utilized at rates of ~1.0–1.1 g/min, due to rate limits in the capacities of the glucose transporter type-2 (GLUT-2), glucose transporter type-5 (GLUT-5), and/or sodium–glucose linked transporter-1 (SGLT-1) carriers in the intestinal membrane [59]. In people with type 1 diabetes, carbohydrate ingestion in readiness for exercise increases blood glucose with the overall magnitude dependent on factors such as amount, digestibility, and glycemic index [6]. This new glucose appearance into body fluid compartments alters body fluid osmolarity.

## 5. Interstitial Glucose Responses to Different Forms of Exercise

Continuous glucose monitoring systems use an enzymatic technique that reacts with circulating glucose in the interstitium [60]. This reaction releases one electron for each glucose molecule and transmits it to an electrode. Within this electrode, an electric current is generated that is passed from a transmitter (attached or incorporated) to a reader (e.g., mobile phone) to reflect the interstitial glucose levels [60].

Data on head-to-head comparisons between continuous and flash glucose monitoring systems remain rare. Aberer et al. [61] have shown that the Abbott FreeStyle Libre (Abbott, Alameda, CA, USA) flash glucose monitoring system was the most accurate (median absolute relative differences (MARD) 8.7 ± 5.9%, *n* = 13), followed by Dexcom G4 Platinum (Dexcom, San Diego, CA, USA) (MARD 15.7 ± 14.6%, *n* = 24), and Medtronic MiniMed 640 G (Medtronic MiniMed, Inc., Northridge, CA, USA) (MARD 19.4 ± 13.5%, *n* = 22) during 30 min (2 × 15 with a 5 min rest) of low-intensity continuous exercise. However, it must be mentioned that only thirteen comparison points between blood glucose and interstitial glucose concentrations were available for the flash glucose monitoring system. Unfortunately, in this trial, glucose concentrations were not classified for hypo-, eu-, and hyperglycemia during exercise. Disagreements in absolute glucose values during exercise between different compartments were found in healthy individuals [62] and in patients with type 1 diabetes [63,64,65]. Interstitial glucose concentrations measured via the SEVEN^®^ PLUS CGM (Dexcom, San Diego, CA, USA) system during exercise under euglycemic conditions are higher compared to capillary (finger sticks) and venous blood, and the capillary blood levels seem to be higher than the venous blood levels [62]. In patients with type 1 diabetes performing high-intensity interval exercise and continuous exercise, no clear trend was found in comparing the capillary blood glucose concentration (earlobe) and interstitial glucose concentration when using the Guardian REAL-time system and the Enlite sensor (Medtronic MiniMed, Inc., Northridge, CA, USA) (no classification for glycemic ranges was performed) [63]. However, in a different study comparing the interstitial glucose (G4 Platinum, Dexcom, San Diego, CA, USA) and venous blood glucose concentration, a trend towards lower levels were found in venous blood when exercising within a euglycemic range (blood glucose during high-intensity interval exercise: 7.56 ± 0.21; blood glucose during continuous exercise: 6.71 ± 0.23) [64]. Interestingly, significantly lower glucose concentrations were found in the interstitium compared to the venous system for continuous exercise and for resistance exercise under a euglycemic range when using the CGMS^®^ System Gold^®^ (Medtronic MiniMed, Inc., Northridge, CA, USA) [65].

The duration of exercise seems not to influence the differences in glucose levels in different compartments [63,64,65]. However, it might be that longer-duration exercise and the potential for dehydration could decrease the glucose supply within the interstitium, resulting in lower glucose levels compared to the capillary and venous systems. Furthermore, the lag time in interstitium could be influenced by the intensity of exercise and the amount of circulating insulin. Low insulin reductions combined with high exercise intensities could increase the lag time between the blood and the interstitium, resulting in severe impairments for continuous and flash glucose monitoring systems. The type and mode of exercise seem not to influence continuous glucose monitoring accuracy [63,64,65]. During high-intensity interval exercise and continuous exercise, continuous glucose monitoring technology shows sufficient potential to trace changes in glucose concentrations [63,64]. During resistance exercise, a mean difference of −0.71 mmol/L was found [65]. However, it should be considered that during resistance exercise the site of sensor placement might directly influence its performance. Therefore, it is prudent to place the sensor away from working muscles.

Only a single study assessed continuous glucose monitoring accuracy during exercise under hypoglycemic conditions [66]. When a hypoglycemic alarm was set at 5.5 mmol/L, the system overestimated the interstitial glucose by 1.6 mmol/L. Therefore, a higher alarm level setting is recommended to avoid exercise-induced hypoglycemia.

Future research should clearly evaluate sensor performance under hypo-, eu-, and hyperglycemic ranges during exercise. Additionally, reported sensor performance during exercise needs to be adjusted for any treatments (e.g., carbohydrate ingestion, bolus insulin correction).

## 6. Continuous and Flash Glucose Monitor Performance

Twenty years of intensive research reveal marked improvements in continuous glucose monitoring performance in the reflection of accuracy, precision, and reliability. Since the start of the millennium, MARDs have been halved from 20% to 10% [67,68]. The Dexcom G5^TM^ Mobile continuous glucose monitoring system, which is approved for patients aged two years and older, shows a MARD of 9% for adults and 10% for pediatric patients [68]. Sensor lifetime is 7 days accompanied with 2 calibrations to capillary blood glucose concentration per day. Its predecessor model, the Dexcom G4^®^ system (Dexcom, San Diego, CA, USA), was found to be less accurate with MARDs of 13% for adults and 15% for pediatric patients [69,70,71,72]. Sensor lifetime is also 7 days, accompanied with 2 calibrations to capillary blood glucose concentration per day.

Medtronic’s (Medtronic MiniMed, Inc., Northridge, CA, USA) flagships are the MiniMed 670 G hybrid closed-loop system and 640 G with SmartGuard^®^ (predictive low glucose), as well as the Medtronic Paradigm Minimed Veo^®^ (530 G) with low glucose suspend. Recently, the MARD for the Medtronic 640 G system was found at 9.6%, using the Guardian^TM^ sensor 3 [73]. Predecessor models using Enlite^TM^ sensor technologies resulted in MARDs of 13.6–14.2% for adults and of about 21% for children from 4–14 years for blood glucose levels below 6.7 mmol/L [72,74]. Sensors need to be changed after 6 days, requiring 2 calibrations per day.

A slightly different technology, Abbotts FreeStyle^®^ Libre FGM system (Abbott, Alameda, CA, USA), was also found to track changes in interstitial glucose accurately with a MARD of 10–11.4% [18,72]. This device shows a sensor lifetime of 14 days and is factory-calibrated. The FreeStyle^®^ Navigator II CGM system has a MARD of 12.3% with a sensor lifetime of up to 5 days and needs to be calibrated 3–4 times per day [72].

Notable, a novel analytical framework that enables spectroscopy-based longitudinal tracking of blood glucose without extensive a priori information was investigated by Spegazzini et al. [75]. Using blood glucose monitoring by Raman spectroscopy as an example, it was found that the efficacy of this approach was comparable to conventional calibration methods (35% reduction in error over partial least squares regression when applied to glucose tolerance tests).

## 7. Continuous and Flash Glucose Monitoring Systems’ (Dis) Advantages and Exercise

These commonly used devices reveal different advantages and disadvantages for daily life; furthermore, accuracy during exercise might be divergent to general MARDs (Table 2).

## 8. Continuous and Flash Glucose Monitor Algorithms

As continuous and flash glucose monitoring systems measure glucose within the interstitial fluid, a certain lag time is present for the glucose to diffuse from the blood into the interstitium. To overcome this lag time and to reduce the risk of inadequate therapy decisions (e.g., overdose in bolus insulin correction), several algorithms are incorporated in continuous and flash glucose monitoring systems. Generally, so-called algorithmically “smart sensors” incorporate three software modules that need to work in real time [77,78]:

- denoising the random noise component
- enhancing the accuracy
- predicting the future glucose concentration

As used by Facchinetti et al. [77], an adaptive self-tunable Bayesian smoother is able to automatically estimate the signal-to-noise ratio present in the continuous glucose monitoringdata [79]. For the enhancement of accuracy, a stochastic deconvolution-based re-calibration algorithm is applied, which re-scales the interstitial glucose data using a simple linear regressor whose parameters are re-calculated for each self-measured blood glucose value [78,80]. Especially this or a similar denoising and enhancement algorithm is inwrought in the Dexcom (Dexcom, San Diego, CA, USA) G4 and G5 sensor technology (505 algorithm).

Predicting future glucose concentrations is based on an autoregressive model of order, one based on the findings of Sparacino et al. [81]. For example, the Medtronic 640 G with SmartGuard^®^ (Medtronic MiniMed, Inc., Northridge, CA, USA) uses an algorithm that predicts the decrease of 20 mg/dL within the next 30 min above the pre-defined “low-threshold”. When combined with an insulin pump, it suspends the infusion of insulin before reaching this value and automatically restarts when it predicts that glucose levels will increase by 20 mg·dL^−1^ above the pre-defined low glucose threshold [78].

## 9. Future Directions

### 9.1. Artificial Pancreas

In line with a recent review, a threshold low-glucose suspend of insulin infusion might be the first step towards an artificial pancreas [72]. The future of artificial pancreas devices promises a more accurate mimicking of endogenous responses to glucose fluctuations; achieved via dual hormone closed-loop systems using insulin and glucagon [82,83]. This system is able to reduce significantly hypoglycemic episodes. Intriguingly, the beneficial effects of dual- compared to single hormone closed-loop systems were observed during continuous and interval exercises [84]. In this study, glucagon was given a microboluses to avoid drastic increases in blood glucose concentrations. Glucagon delivery was based on logical rules considering glucose concentration estimates and their trends. Importantly, the insulin delivery algorithm took account of the injected glucagon onboard via the dual-hormone artificial pancreas. During continuous exercise, a total glucagon dose of 0.126 ± 0.057 mg was administered and during interval exercise 0.093 ± 0.068 mg. The split total glucagon dose was delivered every 10 min during the 60 min exercise sessions. Mini-doses of glucagon were found to be efficacious and safe to treat mild hypoglycemia in adults with type 1 diabetes [85]. Successful hypoglycemia-treatment criteria were met for 94% when using a mini-dose of glucagon in comparison to 95% when giving oral glucose tablets.

Instead of glucagon selective antagonism of somatostatin receptor type 2 (SSTR2) might also be reasonable (within the artificial pancreas) [86]. SSTR2 antagonism after recurrent hypoglycemia ameliorates the glucagon and corticosterone responses, as well as decreases the risk of insulin-induced hypoglycemia in rats with type 1 diabetes. However, a safety profile of SSTR2 antagonism will need to be established. Potential adverse effects might be increased gastric acid secretion and effects on pituitary and adrenal hormone secretion [87].

In real life, the only commercially available system (FDA approved), which is similar to an artificial pancreas, is the hybrid closed-loop system from Medtronic (670 G with SmartGuard^®^) (Medtronic MiniMed, Inc., Northridge, CA, USA). However, from the authors’ point of view, the fundamental problem in the development of a “real” artificial pancreas might be the sensor inaccuracy and sensor/interstitial glucose lag time to blood glucose concentration. For example, the Abbott FreeStyle Libre (Abbott, Alameda, CA, USA) flash glucose monitoring system shows an average lag time of 4.5–4.8 min (for both clinic and home-phases) [18]. In a rapidly changing internal environment during acute exercise combined with low doses of circulating bolus insulin this lag time could potentially rise by 200%, as observed in our lab in two ongoing clinical trials (U1111-1174-6676; DRKS00013477). Furthermore, if the artificial pancreas is designated to work autonomously, the use of detectors of physical activity (e.g., heart rate monitor, accelerometer, small spirometry devices) needs to be incorporated from the very early stages of development.

### 9.2. Implantable Sensors

In a recent pivotal study, the MARD of the Eversense implantable continuous glucose monitoring sensor (Senseonics Inc., Germantown, MD, USA) was found at 11.1% against reference glucose values above 4.2 mmol/L [88]. Implantable continuous glucose monitoring systems may provide advantages since frequent sensor insertions are not needed and the transmitter can be removed without sensor replacement. However, the implantation and removal are a minor surgical procedure and accompanied by some discomfort.

## 10. Conclusions

As more health services and insurance companies advocate their use, understanding continuous and flash glucose monitoring systems for its strengths and limitations around exercise may offer more confidence for patients aiming to better manage glycemia. This review detailed the complexities of acute physical exercise and offers an integrated understanding of the efficacy of glucose monitoring with current technology around a heightened metabolic state such as exercise.

## Figures and Tables

**Table 1:** Glucose transport from blood to skeletal muscle (per 110/g) [33,47].

	Rest	Heavy Exercise	Fractional Δ (Exercise/Rest)
Skeletal muscle glucose consumption (*J_s_*), µmol/min	1.4	60	×43
Arterial glucose concentration (*C_a_*), mM	5.0	5.0	-
Venous concentration (*C_v_*), mM	4.4	4.0	×0.9
Extraction *E*, %	11.2	20	×1.8
Blood flow, mL/min	2.5	60	×24
Perfused capillary density, per mm^2^	250	1000	×4
Diffusion capacity (*PS*), cm^3^/min	5	20	×4
Mean concentration difference across capillary wall (*ΔC = J_s_/PS*), mM	0.3	3.0	×10
Mean pericapillary concentration (*Ci*), mM	4.7	2.0	×0.4
Krogh Cylinder radius, µm	36	18	×0.5

**Table 2:** Advantages, disadvantages, and exercise performance in continuous and flash glucose monitoring systems.

	Advantage	Disadvantage	Exercise Performance
Dexcom G5^TM^	Hypo- and hyperglycemia alerts; rise and fall (rate of change) alerts; compatible with mobile devices; online live monitoring with different mobile devices (e.g., for parents), CE ^1^ mark (European Union). Approved for non-adjuvant use; compatible with Apple iPhone 4S and subsequent iOS models	Requires calibration to blood glucose; no integrated bolus wizard	N/A for Dexcom G5^TM^
Dexcom G4^TM^	Hypo- and hyperglycemia alerts; rise and fall (rate of change) alerts; available integrated with the Animas Vibe pump	Requires calibration to blood glucose; no integrated bolus wizard	Continuous exercise: MARD ^2^ 13.6–18.6%; interval exercise: MARD 13.3–17.7% [61,64,76] (interstitial glucose compared to venous plasma glucose)
Medtronic 670 G with SmartGuard^®^	Hybrid closed-loop system when combined with insulin pump (automatic insulin delivery when glucose is high); predictive low glucose-suspend when combined with insulin pump; predictive low glucose alert; hypo- and hyperglycemia alerts; rise and fall (rate of change) alerts; Bluetooth connected to glucometer (CONTOUR, NEXT LINK 2.4); bolus wizard	Requires calibration to blood glucose	N/A ^3^ for this specific system
Medtronic 640 G with SmartGuard^®^	Predictive low glucose-suspend when combined with insulin pump; predictive low glucose alert; hypo- and hyperglycemia alerts; rise and fall (rate of change) alerts; Bluetooth connected to glucometer (CONTOUR, NEXT LINK 2.4); bolus wizard	Requires calibration to blood glucose	Continuous exercise: MARD 19.4% [61] (interstitial glucose compared to venous plasma glucose)
Medtronic Paradigm Minimed^®^ Veo (530 G)	Low-glucose-suspend when combined with insulin pump; hypo- and hyperglycemia alerts; rise and fall (rate of change) alerts; Bluetooth connected to glucometer (CONTOUR, NEXT LINK 2.4); bolus wizard;	Requires calibration to blood glucose	Continuous exercise: MARD 12.8–23.7%; interval exercise: MARD 15.5–26.5% [63] (interstitial glucose compared to capillary blood glucose)
FreeStyle^®^ Libre Flash glucose monitoring	Factory-calibrated; long sensor lifetime (14 days); integrated glucometer; integrated blood ketone measurement; cheap sensor costs; integrated bolus wizard	No automatic hypo- or hyperglycemia alerts; not combinable with pump	Continuous exercise: MARD 8.7% [61] (interstitial glucose compared to venous plasma glucose)
FreeStyle^®^ Navigator II CGM system	30 m transmission range; new result every minute; hypo- and hyperglycemia alerts; early warning alarms; integrated glucometer	Fixed time points for calibration: 1, 2, 10, 24, and 72 h after sensor insertion	N/A for the second generation

^1^ CE (Conformité Européenne) marking is a certification mark that indicates conformity with health, safety, and environmental protection standards for products sold within the European Economic Area. ^2^ MARD: Median Absolute Relative Difference. ^3^ N/A: Not Applicable.
